# Heart-to-Heart Café: An Innovative Intervention to Promote Advance Care Planning Among Chinese Americans

**DOI:** 10.1093/geroni/igaf122.3786

**Published:** 2025-12-31

**Authors:** Kaipeng Wang, Yaolin Pei, Xiaoyouxiang Li, Aprille Arena, Jina Park, Leslie Hasche, Sandy Stokes

**Affiliations:** University of Denver, Denver, Colorado, United States; The University of Texas at Austin, Austin, Texas, United States; University of Denver, Denver, Colorado, United States; University of Denver, Denver, Colorado, United States; University of Denver, Denver, Colorado, United States; University of Denver, Denver, Colorado, United States; Chinese American Coalition for Compassionate Care, Cupertino, California, United States

## Abstract

Advance care planning (ACP) is associated with improved end-of-life (EOL) care quality, reduced use of aggressive medical treatments, and lower decisional burden for healthcare surrogates. Older Chinese Americans have a significantly lower completion rate of advance directives (ADs) compared to older Americans in general. Thus, a culturally responsive intervention is needed to promote ACP among Chinese Americans. Using a one-group pretest-posttest mixed methods design, this project aims to study the feasibility of Heart-to-Heart (HTH) Café on attitude and readiness regarding ACP among Chinese American adults with a family member aged 60 or older. The program consists of four sessions: (1) HTH Café card games, (2) legal documents lecture, (3) palliative and hospice care lecture, and (4) AD completion workshop. All sessions were virtually offered in Mandarin over a total of five weeks. We evaluated the program’s feasibility using a mixed-methods approach, incorporating pre-post surveys, focus groups, and individual interviews. Seventeen participants were successfully enrolled in the program, and 15 (88%) attended at least three sessions and completed the post-test survey. The results of paired t-tests showed statistically significant improvements in knowledge of ACP and palliative care, as well as a decrease in fear of death. While supporting these results, qualitative findings from thematic analysis further revealed the high feasibility of the program, participants’ improved knowledge of ACP, and enhanced readiness for discussions about death and ACP. Findings highlight the feasibility of a culturally innovative group approach and underscore the need for continued community outreach in ACP promotion among Chinese Americans.

